# Correspondence

**Published:** 1865-01

**Authors:** Charles A. Lee

**Affiliations:** Peekskill


### CORRESPONDENCE.

For the Buffalo Medical and Surgical Journal.

PEEKSKILL, December 30, 1864.

J. F. MINER, M. D., Editor Medical and Surgical Journal:

Dear Sir:—After Dr. Willard's statement in the last number of your Journal I am bound to exonerate him from any intention to do an act of injustice towards the University of Buffalo; while, at the same time, it is due to myself to state, why I deemed it unnecessary, (if not an inadvertence, as it very possibly may have been,) to register myself as a duly appointed delegate from the College, and first, this appeared, as I may have thought sufficiently from my credentials; by which, the registry, if deficient could have been easily corrected. Besides, two days before the meeting of the State Society, I wrote to Dr. Willard from Buffalo, enclosing the resolution already referred to, and requesting him to offer it, in case I should not be able to attend myself as a delegate from the College. Finding I could attend, I telegraphed Dr. W. the day previous to the meeting, saying he need not offer the resolution, as I expected to be present. I did attend, and offered the same in behalf of the Faculty of the University; which, after pretty full discussion, was unanimously adopted. I saw Dr. W. frequently and conversed with him as often; he knowing well that I had just come from Buffalo, and was a member of the Medical Faculty of the College, and acting in their behalf. I had therefore every reason to suppose he was aware I represented the institution, even if I had presented no credentials nor registered my name as their delegate.

Still, if the registry is to be recognized as the only valid proof of delegation, and it be not allowable to rely on other evidence, which would seem perhaps equally satisfactory, it is well that the fact should be understood, in order that delegates hereafter may not be as careless, as it seems, I have been.

CHARLES A. LEE.

